# Supplementary material for: An RNAi screen unravels the complexities of Rho GTPase networks in skin morphogenesis
Source: eLife. 2019 Sep 25;8:e50226. doi: 10.7554/eLife.50226 (PMC6768663; doi:10.7554/eLife.50226)
Supplement: Supplementary file 3. [file elife-50226-supp3.docx]

|  | **Supplementary File 3.** Genes With ≥ Two shRNAs Showing an Absolute Enrichment or Depletion in The Hair Follicle Fraction | | | | | | | |
| --- | --- | --- | --- | --- | --- | --- | --- | --- |
| Candidate | | Gene | Fraction shRNA | Mean  *in vitro* | Mean E18.5 HF | Fold change | *P* value | q value |
| Rho GTPases | | |  |  |  |  |  |  |
| 1 | | *Rhou* TRCN0000287360 | 3 of 9 | 361.7 | 1731 | 4.785734034 | 2.56432E-05 | 0.001281156 |
|  | | *Rhou* TRCN0000077506 |  | 1654 | 4142 | 2.504232164 | 0.007898817 | 0.022724639 |
|  | | *Rhou* TRCN0000077505 |  | 635.8 | 1548 | 2.434727902 | 0.004731611 | 0.016449743 |
| 2 | | *Rhoj* TRCN0000312386 | 2 of 8 | 42.39 | 141.7 | 3.342769521 | 0.000112373 | 0.002041057 |
|  | | *Rhoj* TRCN0000312323 |  | 267.7 | 586.7 | 2.191632424 | 0.018395836 | 0.041766056 |
| 3 | | *Rhob* TRCN0000077533 | 2 of 5 | 104333 | 47399 | 0.454304966 | 0.004463716 | 0.015827519 |
|  | | *Rhob* TRCN0000077534 |  | 53638 | 9107 | 0.169786346 | 0.001368599 | 0.007427153 |
| 4 | | *Rac3* TRCN0000065396 | 2 of 5 | 6251 | 2539 | 0.406175012 | 0.002363527 | 0.010320085 |
|  | | *Rac3* TRCN0000065393 |  | 71947 | 27482 | 0.381975621 | 0.012764056 | 0.032181331 |
| 5 | | *Rac1* TRCN0000055190 | 3 of 9 | 35603 | 13614 | 0.382383507 | 0.00649188 | 0.020046654 |
|  | | *Rac1* TRCN0000055192 |  | 52705 | 15837 | 0.300483825 | 0.008017457 | 0.022724639 |
|  | | *Rac1* TRCN0000055189 |  | 7963 | 1300 | 0.163255055 | 8.49622E-05 | 0.00180039 |
| 6 | | *Rhov* TRCN0000077672 | 3 of 5 | 5876 | 1810 | 0.308032675 | 0.005055775 | 0.017142565 |
|  | | *Rhov* TRCN0000077669 |  | 1291 | 371.5 | 0.287761425 | 0.001566945 | 0.007969036 |
|  | | *Rhov* TRCN0000077670 |  | 23933 | 3819 | 0.159570468 | 0.017027654 | 0.039465135 |
| 7 | | *Rhoa* TRCN0000068202 | 2 of 10 | 3633 | 1008 | 0.277456647 | 6.4617E-05 | 0.001554302 |
|  | | *Rhoa* TRCN0000068201 |  | 80.4 | 22.14 | 0.275373134 | 0.01086263 | 0.028848828 |
| RhoGEFs | |  |  |  |  |  |  |  |
| 8 | | *Arhgef17* TRCN0000267420 | 2 of 5 | 1752 | 880 | 0.502283105 | 0.002943582 | 0.01176389 |
|  | | *Arhgef17* TRCN0000252551 |  | 2083 | 930.2 | 0.446567451 | 0.017706819 | 0.040616157 |
| 9 | | *Arhgef40* TRCN0000121408 | 2 of 5 | 13354 | 6659 | 0.498652089 | 0.000265726 | 0.003093146 |
|  | | *Arhgef40* TRCN0000121410 |  | 1889 | 500.1 | 0.26474325 | 0.000577728 | 0.004357437 |
| 10 | | *Arhgef3* TRCN0000110049 | 3 of 5 | 14521 | 7214 | 0.496797741 | 0.001121287 | 0.006438355 |
|  | | *Arhgef3* TRCN0000110047 |  | 13573 | 6043 | 0.445222132 | 0.002153773 | 0.009779889 |
|  | | *Arhgef3* TRCN0000110046 |  | 7497 | 2913 | 0.388555422 | 0.03905341 | 0.07348316 |
| 11 | | *Prex1* TRCN0000247271 | 3 of 5 | 432.3 | 211.3 | 0.488780939 | 0.017277536 | 0.039940279 |
|  | | *Prex1* TRCN0000247272 |  | 170.4 | 41.87 | 0.245715962 | 0.000331545 | 0.003191108 |
|  | | *Prex1* TRCN0000247273 |  | 565.6 | 226.5 | 0.400459689 | 0.020381513 | 0.044433398 |
| 12 | | *Arhgef5* TRCN0000252188 | 3 of 5 | 202.3 | 98.8 | 0.488383589 | 0.030415994 | 0.060977107 |
|  | | *Arhgef5* TRCN0000252190 |  | 302.6 | 42.28 | 0.139722406 | 0.007177372 | 0.021292872 |
|  | | *Arhgef5* TRCN0000252191 |  | 705 | 204 | 0.289361702 | 0.019555523 | 0.043294566 |
| 13 | | *Arhgef1* TRCN0000110053 | 3 of 5 | 18473 | 8991 | 0.486710334 | 0.004243924 | 0.015108369 |
|  | | *Arhgef1* TRCN0000110054 |  | 35669 | 6249 | 0.175194146 | 0.000544886 | 0.004191829 |
|  | | *Arhgef1* TRCN0000110050 |  | 94.42 | 24.84 | 0.263079856 | 0.017562982 | 0.040390321 |
| 14 | | *Dock2* TRCN0000091247 | 2 of 5 | 2971 | 1445 | 0.486368226 | 0.009792336 | 0.026438881 |
|  | | *Dock2* TRCN0000091246 |  | 12116 | 4060 | 0.33509409 | 0.00078306 | 0.005162396 |
| 15 | | *Prex2* TRCN0000081352 | 3 of 5 | 55654 | 26720 | 0.480109246 | 0.007154654 | 0.021292872 |
|  | | *Prex2* TRCN0000081348 |  | 42731 | 9350 | 0.218810699 | 0.000323951 | 0.003191108 |
|  | | *Prex2* TRCN0000081350 |  | 23062 | 7559 | 0.327768624 | 5.34842E-05 | 0.001355019 |
| 16 | | *Tiam2* TRCN0000110006 | 3 of 6 | 4469 | 1949 | 0.436115462 | 0.024126541 | 0.050642975 |
|  | | *Tiam2* TRCN0000110005 |  | 34165 | 14332 | 0.419493634 | 0.000988259 | 0.006070629 |
|  | | *Tiam2* TRCN0000110009 |  | 71738 | 14199 | 0.197928573 | 0.001470095 | 0.007606888 |
| 17 | | *Arhgef9* TRCN0000226313 | 2 of 10 | 1421 | 617.8 | 0.434764251 | 0.016007088 | 0.037688646 |
|  | | *Arhgef9* TRCN0000258240 |  | 227.9 | 33.85 | 0.148530057 | 0.02090024 | 0.045148577 |
| 18 | | *Mcf2l* TRCN0000110012 | 4 of 5 | 2888 | 1249 | 0.432479224 | 0.000368193 | 0.003310811 |
|  | | *Mcf2l* TRCN0000110013 |  | 7606 | 477.1 | 0.062726795 | 3.87464E-05 | 0.001281156 |
|  | | *Mcf2l* TRCN0000110010 |  | 62227 | 25114 | 0.403586867 | 0.007342903 | 0.02163968 |
|  | | *Mcf2l* TRCN0000110011 |  | 15824 | 3891 | 0.245892315 | 7.46817E-05 | 0.00162114 |
| 19 | | *Mcf2* TRCN0000042654 | 2 of 6 | 17131 | 7327 | 0.427704162 | 0.00675353 | 0.020584389 |
|  | | *Mcf2* TRCN0000174066 |  | 88.38 | 31.2 | 0.353021045 | 0.000277004 | 0.003093146 |
| 20 | | *Arhgef15* TRCN0000012883 | 2 of 12 | 17156 | 6896 | 0.401958498 | 0.000683814 | 0.004754644 |
|  | | *Arhgef15* TRCN0000175834 |  | 5427 | 1682 | 0.309931822 | 8.79433E-05 | 0.001820222 |
| 21 | | *Abr* TRCN0000105838 | 3 of 5 | 3900 | 1558 | 0.399487179 | 0.000349846 | 0.003243364 |
|  | | *Abr* TRCN0000105839 |  | 314.9 | 28.77 | 0.091362337 | 0.002968826 | 0.01176389 |
|  | | *Abr* TRCN0000105837 |  | 37428 | 13386 | 0.357646682 | 0.000514875 | 0.004075792 |
| 22 | | *Fgd2* TRCN0000110044 | 2 of 4 | 20446 | 7706 | 0.376895236 | 0.030506741 | 0.061013482 |
|  | | *Fgd2* TRCN0000110041 |  | 116.3 | 43.56 | 0.374548581 | 0.006389555 | 0.019814298 |
| 23 | | *Dnmbp* TRCN0000329046 | 2 of 8 | 1289 | 483.4 | 0.375019395 | 0.048523147 | 0.086892558 |
|  | | *Dnmbp* TRCN0000329105 |  | 1110 | 392.6 | 0.353693694 | 0.005768736 | 0.018468255 |
| 24 | | *Als2* TRCN0000041030 | 2 of 5 | 76865 | 27060 | 0.352045795 | 0.001432711 | 0.007500664 |
|  | | *Als2* TRCN0000041032 |  | 40998 | 6743 | 0.164471438 | 0.000180149 | 0.002419948 |
| 25 | | *Dock10* TRCN0000251515 | 2 of 5 | 4730 | 1624 | 0.343340381 | 0.00015923 | 0.002285726 |
|  | | *Dock10* TRCN0000251513 |  | 288.6 | 31.93 | 0.110637561 | 0.006105942 | 0.019270525 |
| 26 | | *Fgd5* TRCN0000110066 | 2 of 5 | 11914 | 3927 | 0.329612221 | 0.008253057 | 0.02331816 |
|  | | *Fgd5* TRCN0000110067 |  | 19287 | 5904 | 0.306112926 | 0.000332443 | 0.003191108 |
| 27 | | *Dock11* TRCN0000217207 | 2 of 3 | 30857 | 9835 | 0.318728327 | 0.002974017 | 0.01176389 |
|  | | *Dock11* TRCN0000200778 |  | 19255 | 3378 | 0.175434952 | 0.000168043 | 0.002369345 |
| 28 | | *Trio* TRCN0000254106 | 2 of 5 | 1095 | 320.4 | 0.29260274 | 0.002257137 | 0.010113594 |
|  | | *Trio* TRCN0000254107 |  | 756.5 | 113.1 | 0.149504296 | 0.001898443 | 0.00898731 |
| 29 | | *Plekhg3* TRCN0000179378 | 3 of 8 | 5385 | 1512 | 0.280779944 | 0.001170606 | 0.006635921 |
|  | | *Plekhg3* TRCN0000183787 |  | 62132 | 9516 | 0.153157793 | 0.001880451 | 0.00898226 |
|  | | *Plekhg3* TRCN0000184514 |  | 5512 | 298.5 | 0.054154572 | 3.74896E-05 | 0.001281156 |
| 30 | | *Plekhg1* TRCN0000251310 | 2 of 5 | 5960 | 1669 | 0.280033557 | 0.014826608 | 0.03585783 |
|  | | *Plekhg1* TRCN0000251309 |  | 625.9 | 130.9 | 0.20913884 | 0.002734308 | 0.011163 |
| 31 | | *Dock9* TRCN0000253067 | 2 of 5 | 260.2 | 68.29 | 0.26245196 | 0.050066369 | 0.088763084 |
|  | | *Dock9* TRCN0000253069 |  | 161.5 | 15.32 | 0.094860681 | 0.002729645 | 0.011163 |
| 32 | | *Arhgef28* TRCN0000110063 | 3 of 5 | 6894 | 1784 | 0.258775747 | 0.000284986 | 0.003093146 |
|  | | *Arhgef28* TRCN0000110064 |  | 66203 | 16504 | 0.249293839 | 0.005444154 | 0.017794787 |
|  | | *Arhgef28* TRCN0000110061 |  | 15288 | 1398 | 0.09144427 | 0.000131847 | 0.002162559 |
| 33 | | *Arhgef11* TRCN0000110183 | 2 of 5 | 20796 | 5182 | 0.249182535 | 0.001190238 | 0.006662336 |
|  | | *Arhgef11* TRCN0000110181 |  | 9941 | 2346 | 0.235992355 | 0.000276589 | 0.003093146 |
| 34 | | *Arhgef2* TRCN0000109986 | 2 of 5 | 39822 | 8201 | 0.205941439 | 0.001860861 | 0.00895225 |
|  | | *Arhgef2* TRCN0000109989 |  | 10908 | 2136 | 0.195819582 | 0.000597593 | 0.004395522 |
| 35 | | *Net1* TRCN0000110083 | 3 of 5 | 1891 | 267 | 0.141195135 | 0.002565212 | 0.01082009 |
|  | | *Net1* TRCN0000110082 |  | 30674 | 3057 | 0.099660951 | 0.000855929 | 0.005441262 |
|  | | *Net1* TRCN0000110081 |  | 20549 | 2018 | 0.098204292 | 7.39004E-05 | 0.00162114 |
| RhoGAPs | |  |  |  |  |  |  |  |
| 36 | | *Grlf1* TRCN0000174933 | 2 of 9 | 6002 | 24777 | 4.128123959 | 0.000551061 | 0.004191829 |
|  | | *Grlf1* TRCN0000216719 |  | 178.8 | 394.1 | 2.204138702 | 0.018667101 | 0.042166803 |
| 37 | | *Arhgap36* TRCN0000283769 | 2 of 5 | 336.1 | 909.6 | 2.7063374 | 0.009854424 | 0.026487763 |
|  | | *Arhgap36* TRCN0000283770 |  | 143.8 | 318.4 | 2.21418637 | 0.004888081 | 0.016861985 |
| 38 | | *Arhgap8* TRCN0000097337 | 2 of 5 | 80511 | 37336 | 0.463737874 | 0.032237614 | 0.063197084 |
|  | | *Arhgap8* TRCN0000097338 |  | 6330 | 465.3 | 0.073507109 | 3.15438E-05 | 0.001281156 |
| 39 | | *Chn2* TRCN0000112401 | 3 of 5 | 30157 | 13877 | 0.460158504 | 0.011012822 | 0.02899826 |
|  | | *Chn2* TRCN0000112402 |  | 23475 | 3684 | 0.156932907 | 1.70826E-05 | 0.001281156 |
|  | | *Chn2* TRCN0000112404 |  | 22718 | 8017 | 0.35289198 | 0.000461641 | 0.003839815 |
| 40 | | *Arhgap22* TRCN0000181968 | 3 of 6 | 145.6 | 62.19 | 0.427129121 | 0.013121315 | 0.032895691 |
|  | | *Arhgap22* TRCN0000197662 |  | 29360 | 11708 | 0.398773842 | 0.001911744 | 0.009002394 |
|  | | *Arhgap22* TRCN0000182379 |  | 908.9 | 148.1 | 0.162944218 | 0.002317764 | 0.010314051 |
| 41 | | *Arhgap4* TRCN0000071699 | 2 of 5 | 18050 | 7635 | 0.42299169 | 0.003728749 | 0.013955361 |
|  | | *Arhgap4* TRCN0000071700 |  | 53168 | 5430 | 0.1021291 | 0.002147874 | 0.009779889 |
| 42 | | *Arhgap23* TRCN0000179417 | 2 of 10 | 38523 | 15498 | 0.402305116 | 0.001189521 | 0.006662336 |
|  | | *Arhgap23* TRCN0000184726 |  | 3653 | 1359 | 0.372022995 | 0.001576208 | 0.007970597 |
| 43 | | *Arap3* TRCN0000288345 | 3 of 9 | 216.4 | 86.58 | 0.400092421 | 0.012103663 | 0.030954771 |
|  | | *Arap3* TRCN0000106139 |  | 25110 | 9086 | 0.361847869 | 0.0070763 | 0.021205075 |
|  | | *Arap3* TRCN0000295613 |  | 482.2 | 65.79 | 0.136437163 | 0.003711929 | 0.013955361 |
| 44 | | *Abr* TRCN0000105838 | 3 of 5 | 3900 | 1558 | 0.399487179 | 0.000349846 | 0.003243364 |
|  | | *Abr* TRCN0000105839 |  | 314.9 | 28.77 | 0.091362337 | 0.002968826 | 0.01176389 |
|  | | *Abr* TRCN0000105837 |  | 37428 | 13386 | 0.357646682 | 0.000514875 | 0.004075792 |
| 45 | | *Depdc1b* TRCN0000173585 | 3 of 5 | 282.2 | 108.3 | 0.383770376 | 0.039852366 | 0.074202104 |
|  | | *Depdc1b* TRCN0000176211 |  | 11569 | 4256 | 0.367879678 | 0.001406346 | 0.007500664 |
|  | | *Depdc1b* TRCN0000193930 |  | 10788 | 2029 | 0.188079347 | 0.003668142 | 0.013892112 |
| 46 | | *Gmip* TRCN0000028244 | 2 of 5 | 286.4 | 108.6 | 0.379189944 | 0.015504892 | 0.037095038 |
|  | | *Gmip* TRCN0000028298 |  | 12441 | 1435 | 0.115344426 | 6.42963E-06 | 0.001281156 |
| 47 | | *Arhgap30* TRCN0000105728 | 2 of 5 | 27688 | 10268 | 0.370846576 | 0.000104014 | 0.002021364 |
|  | | *Arhgap30* TRCN0000105727 |  | 29230 | 8793 | 0.300821074 | 1.61793E-05 | 0.001281156 |
| 48 | | *Chn1* TRCN0000112395 | 2 of 5 | 44355 | 16051 | 0.361875775 | 0.001424229 | 0.007500664 |
|  | | *Chn1* TRCN0000112396 |  | 4065 | 709.4 | 0.174514145 | 1.59982E-05 | 0.001281156 |
| 49 | | *Stard13* TRCN0000106252 | 2 of 5 | 33023 | 11916 | 0.360839415 | 0.000395262 | 0.003463678 |
|  | | *Stard13* TRCN0000106254 |  | 64164 | 18709 | 0.291580949 | 0.000839162 | 0.005401014 |
| 50 | | *Tagap* TRCN0000097230 | 2 of 5 | 27106 | 9684 | 0.357264074 | 0.003595982 | 0.01368362 |
|  | | *Tagap* TRCN0000097229 |  | 18031 | 3407 | 0.18895236 | 3.51573E-05 | 0.001281156 |
| 51 | | *Ocrl* TRCN0000080957 | 3 of 5 | 18492 | 6596 | 0.356694787 | 0.003098508 | 0.012148335 |
|  | | *Ocrl* TRCN0000080954 |  | 13381 | 4406 | 0.32927285 | 0.001053156 | 0.006367698 |
|  | | *Ocrl* TRCN0000080956 |  | 31753 | 5924 | 0.186565049 | 0.000111767 | 0.002041057 |
| 52 | | *Arhgap28* TRCN0000195897 | 2 of 4 | 38371 | 13150 | 0.342706732 | 0.000238387 | 0.002934337 |
|  | | *Arhgap28* TRCN0000179210 |  | 3787 | 685.1 | 0.180908371 | 0.006280678 | 0.019613345 |
| 53 | | *Arhgap15* TRCN0000197579 | 2 of 8 | 12542 | 4279 | 0.341173657 | 0.001923513 | 0.00901014 |
|  | | *Arhgap15* TRCN0000176672 |  | 63607 | 2472 | 0.038863647 | 0.001737919 | 0.00858017 |
| 54 | | *Depdc7* TRCN0000191595 | 2 of 4 | 9004 | 3064 | 0.340293203 | 0.014811764 | 0.03585783 |
|  | | *Depdc7* TRCN0000200619 |  | 56502 | 8383 | 0.14836643 | 0.00017695 | 0.002419948 |
| 55 | | *Dlc1* TRCN0000251077 | 2 of 8 | 1173 | 389.8 | 0.332310315 | 0.00235013 | 0.010320085 |
|  | | *Dlc1* TRCN0000217664 |  | 4490 | 1264 | 0.281514477 | 0.000319052 | 0.003191108 |
| 56 | | *Arhgap11a* TRCN0000193496 | 2 of 4 | 48199 | 15315 | 0.317745181 | 0.005449449 | 0.017794787 |
|  | | *Arhgap11a* TRCN0000193495 |  | 5506 | 775.6 | 0.140864511 | 1.77487E-05 | 0.001281156 |
| 57 | | *Pik3r2* TRCN0000025088 | 2 of 9 | 1091 | 335.7 | 0.307699358 | 0.005084986 | 0.017142565 |
|  | | *Pik3r2* TRCN0000025086 |  | 6190 | 1439 | 0.232471729 | 0.000326435 | 0.003191108 |
| 58 | | *Arhgap31* TRCN0000105801 | 2 of 5 | 1883 | 574.7 | 0.305204461 | 0.000396961 | 0.003463678 |
|  | | *Arhgap31* TRCN0000105802 |  | 12700 | 1982 | 0.156062992 | 3.11994E-05 | 0.001281156 |
| 59 | | *Arhgap19* TRCN0000192761 | 2 of 5 | 14889 | 4525 | 0.303915642 | 0.000242884 | 0.002934337 |
|  | | *Arhgap19* TRCN0000191107 |  | 49686 | 14551 | 0.292859155 | 5.17117E-05 | 0.001355019 |
| 60 | | *Arhgap20* TRCN0000097342 | 2 of 5 | 2840 | 457.2 | 0.160985915 | 0.000247809 | 0.002940666 |
|  | | *Arhgap20* TRCN0000097343 |  | 9550 | 1290 | 0.135078534 | 3.92899E-06 | 0.001281156 |
| RhoGDIs | |  |  |  |  |  |  |  |
| 61 | | *Arhgdib* TRCN0000106180 | 4 of 5 | 52693 | 16563 | 0.314330177 | 0.004234133 | 0.015108369 |
|  | | *Arhgdib* TRCN0000106182 |  | 3855 | 840.9 | 0.218132296 | 1.36004E-05 | 0.001281156 |
|  | | *Arhgdib* TRCN0000106181 |  | 71690 | 8120 | 0.113265448 | 0.000136278 | 0.002162559 |
|  | | *Arhgdib* TRCN0000106183 |  | 19665 | 1880 | 0.095601322 | 3.88665E-05 | 0.001281156 |
