# Supplementary material for: An RNAi screen unravels the complexities of Rho GTPase networks in skin morphogenesis
Source: eLife. 2019 Sep 25;8:e50226. doi: 10.7554/eLife.50226 (PMC6768663; doi:10.7554/eLife.50226)
Supplement: Supplementary file 4. [file elife-50226-supp4.docx]

**Supplementary File 4.** Genes With ≥ Two shRNAs Showing an Absolute Enrichment or Depletion in the Epidermal Fraction.

| Candidate | Gene | Fraction  shRNA | Mean *in vitro* | Mean E18.5 Epi | Fold change | *P* value | q value |
| --- | --- | --- | --- | --- | --- | --- | --- |
| RhoGTPases |  |  |  |  |  |  |  |
| 1 | *Rhoj* TRCN0000312323 | 2 of 8 | 267.7 | 672.6 | 2.512514008 | 0.003209203 | 0.021026456 |
|  | *Rhoj* TRCN0000312386 |  | 42.39 | 103.2 | 2.434536447 | 0.001216856 | 0.01212366 |
| 2 | *Rac2* TRCN0000065344 | 2 of 5 | 32985 | 15838 | 0.480157647 | 0.01844816 | 0.067015766 |
|  | *Rac2* TRCN0000065343 |  | 30373 | 4986 | 0.164158957 | 0.000796112 | 0.00970602 |
| 3 | *Rhov* TRCN0000077672 | 2 of 5 | 5876 | 2806 | 0.477535739 | 0.024316705 | 0.081055683 |
|  | *Rhov* TRCN0000077669 |  | 1291 | 367.2 | 0.284430674 | 0.008539153 | 0.040277556 |
| 4 | *Rhoa* TRCN0000068201 | 2 of 10 | 80.4 | 26.32 | 0.327363184 | 0.007886691 | 0.038147583 |
|  | *Rhoa* TRCN0000068202 |  | 3633 | 947.6 | 0.260831269 | 0.000128237 | 0.003153132 |
| RhoGEFs |  |  |  |  |  |  |  |
| 5 | *Prex2* TRCN0000081350 | 2 of 5 | 23062 | 11598 | 0.502905212 | 0.002708921 | 0.018962533 |
|  | *Prex2* TRCN0000081348 |  | 42731 | 15506 | 0.362874728 | 0.007118859 | 0.035689472 |
| 6 | *Tiam2* TRCN0000110005 | 2 of 6 | 34165 | 16850 | 0.49319479 | 0.003313985 | 0.021528809 |
|  | *Tiam2* TRCN0000110009 |  | 71738 | 21564 | 0.300593828 | 0.003004383 | 0.020195315 |
| 7 | *Itsn1* TRCN0000026163 | 4 of 13 | 6679 | 3228 | 0.483305884 | 0.001041691 | 0.010832025 |
|  | *Itsn1* TRCN0000328078 |  | 4168 | 1493 | 0.358205374 | 0.008182611 | 0.039153356 |
|  | *Itsn1* TRCN0000111565 |  | 17423 | 4957 | 0.284508982 | 0.000602451 | 0.008123959 |
|  | *Itsn1* TRCN0000026190 |  | 20659 | 1931 | 0.093470158 | 8.95326E-05 | 0.00301123 |
| 8 | *Mcf2l* TRCN0000110012 | 3 of 5 | 2888 | 1374 | 0.475761773 | 0.005966508 | 0.031608288 |
|  | *Mcf2l* TRCN0000110011 |  | 15824 | 4193 | 0.26497725 | 0.000115443 | 0.00301123 |
|  | *Mcf2l* TRCN0000110013 |  | 7606 | 1116 | 0.146726269 | 0.000101969 | 0.00301123 |
| 9 | *Abr* TRCN0000105837 | 3 of 5 | 37428 | 17483 | 0.467110185 | 0.003365283 | 0.021703635 |
|  | *Abr* TRCN0000105839 |  | 314.9 | 32.84 | 0.104287075 | 0.004350246 | 0.026072305 |
|  | *Abr* TRCN0000105838 |  | 3900 | 1506 | 0.386153846 | 0.001559512 | 0.013966164 |
| 10 | *Arhgef11* TRCN0000110183 | 2 of 5 | 20796 | 9666 | 0.464800923 | 0.009376752 | 0.043017057 |
|  | *Arhgef11* TRCN0000110181 |  | 9941 | 2706 | 0.272206015 | 0.000517319 | 0.007193966 |
| 11 | *Fgd5* TRCN0000110067 | 2 of 5 | 19287 | 8639 | 0.447918287 | 0.001589311 | 0.014004816 |
|  | *Fgd5* TRCN0000110066 |  | 11914 | 5203 | 0.436713111 | 0.011008942 | 0.047562904 |
| 12 | *Arhgef37* TRCN0000110204 | 3 of 5 | 5162 | 2277 | 0.441108098 | 0.026355509 | 0.086236776 |
|  | *Arhgef37* TRCN0000110202 |  | 30796 | 5815 | 0.188823224 | 0.000172864 | 0.003344541 |
|  | *Arhgef37* TRCN0000110203 |  | 12521 | 4606 | 0.367861992 | 0.029444547 | 0.094264918 |
| 13 | *Arhgef15* TRCN0000012883 | 2 of 12 | 17156 | 7477 | 0.435824201 | 0.013106447 | 0.054279323 |
|  | *Arhgef15* TRCN0000175834 |  | 5427 | 1911 | 0.352128248 | 0.000820305 | 0.009721583 |
| 14 | *Dock7* TRCN0000217351 | 4 of 8 | 28400 | 12374 | 0.435704225 | 0.013341089 | 0.054970227 |
|  | *Dock7* TRCN0000191485 |  | 25052 | 4488 | 0.179147373 | 8.57106E-05 | 0.00301123 |
|  | *Dock7* TRCN0000215569 |  | 22625 | 3304 | 0.146033149 | 0.000151043 | 0.003153132 |
|  | *Dock7* TRCN0000244527 |  | 2859 | 757.8 | 0.265057712 | 0.003455553 | 0.021967445 |
| 15 | *Fgd1* TRCN0000110022 | 2 of 9 | 2804 | 1170 | 0.417261056 | 0.016218801 | 0.061686892 |
|  | *Fgd1* TRCN0000110020 |  | 87488 | 18393 | 0.210234546 | 0.001705106 | 0.014416243 |
| 16 | *Ngef* TRCN0000176123 | 2 of 8 | 5530 | 2132 | 0.385533454 | 0.006049905 | 0.031860447 |
|  | *Ngef* TRCN0000215945 |  | 14668 | 3924 | 0.267521134 | 0.003862011 | 0.024036294 |
| 17 | *Arhgef5* TRCN0000252188 | 3 of 5 | 202.3 | 69.78 | 0.344933267 | 0.001282071 | 0.012402645 |
|  | *Arhgef5* TRCN0000252191 |  | 705 | 181.3 | 0.257163121 | 0.013796829 | 0.055570967 |
|  | *Arhgef5* TRCN0000252190 |  | 302.6 | 46.51 | 0.153701256 | 0.009150733 | 0.04219768 |
| 18 | *Arhgef17* TRCN0000267420 | 2 of 5 | 1752 | 561.4 | 0.32043379 | 0.00104669 | 0.010832025 |
|  | *Arhgef17* TRCN0000252551 |  | 2083 | 588.9 | 0.282717235 | 0.005879182 | 0.031332166 |
| 19 | *Arhgef2* TRCN0000109986 | 2 of 5 | 39822 | 11608 | 0.291497162 | 0.004627275 | 0.026742043 |
|  | *Arhgef2* TRCN0000109989 |  | 10908 | 3023 | 0.277136047 | 0.001986981 | 0.015512398 |
| 20 | *Arhgef28* TRCN0000110063 | 2 of 5 | 6894 | 1755 | 0.254569191 | 0.000485864 | 0.006974497 |
|  | *Arhgef28* TRCN0000110061 |  | 15288 | 1951 | 0.127616431 | 0.00022737 | 0.003967826 |
| 21 | *Plekhg3* TRCN0000179378 | 3 of 5 | 5385 | 1284 | 0.238440111 | 0.000979588 | 0.010660671 |
|  | *Plekhg3* TRCN0000183787 |  | 62132 | 14374 | 0.231346166 | 0.003017952 | 0.020195315 |
|  | *Plekhg3* TRCN0000184514 |  | 5512 | 286.2 | 0.051923077 | 4.99714E-05 | 0.00301123 |
| 22 | *Net1* TRCN0000110082 | 3 of 5 | 30674 | 6661 | 0.217154593 | 0.002600394 | 0.018664116 |
|  | *Net1* TRCN0000110083 |  | 1891 | 232.1 | 0.122739291 | 0.00231057 | 0.017280731 |
|  | *Net1* TRCN0000110081 |  | 20549 | 2037 | 0.099128911 | 7.45771E-05 | 0.00301123 |
| 23 | *Dock9* TRCN0000253067 | 2 of 5 | 260.2 | 38.76 | 0.148962337 | 0.025236486 | 0.083496179 |
|  | *Dock9* TRCN0000253069 |  | 161.5 | 15.58 | 0.096470588 | 0.004510536 | 0.026237756 |
| RhoGAPs |  |  |  |  |  |  |  |
| 24 | *Grlf1* TRCN0000174933 | 3 of 9 | 6002 | 34280 | 5.711429523 | 0.000670209 | 0.008368099 |
|  | *Grlf1* TRCN0000216719 |  | 178.8 | 639.7 | 3.577740492 | 0.001663868 | 0.014377416 |
|  | *Grlf1* TRCN0000174015 |  | 7241 | 20110 | 2.777240713 | 0.000279727 | 0.004494796 |
| 25 | *Arhgap4* TRCN0000071698 | 2 of 5 | 9720 | 4610 | 0.474279835 | 0.000111582 | 0.00301123 |
|  | *Arhgap4* TRCN0000071700 |  | 53168 | 6839 | 0.128630003 | 0.002716802 | 0.018962533 |
| 26 | *Arhgap30* TRCN0000105728 | 2 of 5 | 27688 | 12981 | 0.468831263 | 0.00051005 | 0.007193966 |
|  | *Arhgap30* TRCN0000105727 |  | 29230 | 9849 | 0.336948341 | 5.28489E-05 | 0.00301123 |
| 27 | *Abr* TRCN0000105837 | 3 of 5 | 37428 | 17483 | 0.467110185 | 0.003365283 | 0.021703635 |
|  | *Abr* TRCN0000105839 |  | 314.9 | 32.84 | 0.104287075 | 0.004350246 | 0.026072305 |
|  | *Abr* TRCN0000105838 |  | 3900 | 1506 | 0.386153846 | 0.001559512 | 0.013966164 |
| 28 | *Dlc1* TRCN0000251077 | 2 of 8 | 1173 | 546 | 0.465473146 | 0.010022035 | 0.045142694 |
|  | *Dlc1* TRCN0000217664 |  | 4490 | 1746 | 0.388864143 | 0.001521103 | 0.013966164 |
| 29 | *Arhgap28* TRCN0000195897 | 2 of 4 | 38371 | 17827 | 0.464595658 | 0.003491992 | 0.02204165 |
|  | *Arhgap28* TRCN0000179210 |  | 3787 | 674.8 | 0.17818854 | 0.00644895 | 0.032986007 |
| 30 | *Arhgap31* TRCN0000105804 | 3 of 5 | 3236 | 1471 | 0.454573548 | 0.00104499 | 0.010832025 |
|  | *Arhgap31* TRCN0000105801 |  | 1883 | 438.8 | 0.233032395 | 0.001081275 | 0.011061321 |
|  | *Arhgap31* TRCN0000105802 |  | 12700 | 2623 | 0.206535433 | 3.44575E-05 | 0.00301123 |
| 31 | *Chn2* TRCN0000112401 | 3 of 5 | 30157 | 13229 | 0.438670955 | 0.021454426 | 0.074009456 |
|  | *Chn2* TRCN0000112402 |  | 23475 | 5454 | 0.232332268 | 0.000143172 | 0.003153132 |
|  | *Chn2* TRCN0000112404 |  | 22718 | 9879 | 0.43485342 | 0.003417069 | 0.021879077 |
| 32 | *Arap1* TRCN0000247787 | 2 of 5 | 584.4 | 247 | 0.422655715 | 0.022804834 | 0.077172251 |
|  | *Arap1* TRCN0000247788 |  | 1339 | 267.8 | 0.2 | 0.002382441 | 0.017523738 |
| 33 | *Arhgap22* TRCN0000181968 | 3 of 6 | 145.6 | 61.28 | 0.420879121 | 0.005804716 | 0.03112167 |
|  | *Arhgap22* TRCN0000182379 |  | 908.9 | 114.2 | 0.125646386 | 0.002510277 | 0.018163794 |
|  | *Arhgap22* TRCN0000197662 |  | 29360 | 11314 | 0.385354223 | 0.019181154 | 0.068012857 |
| 34 | *Arhgap19* TRCN0000192761 | 2 of 5 | 14889 | 6237 | 0.418899859 | 0.00010091 | 0.00301123 |
|  | *Arhgap19* TRCN0000191107 |  | 49686 | 20463 | 0.411846395 | 0.001716991 | 0.014416243 |
| 35 | *Tagap* TRCN0000097230 | 2 of 5 | 27106 | 10306 | 0.380211023 | 0.014783632 | 0.057456035 |
|  | *Tagap* TRCN0000097229 |  | 18031 | 4458 | 0.247240863 | 0.000116489 | 0.00301123 |
| 36 | *Ocrl* TRCN0000080954 | 2 of 5 | 13381 | 4725 | 0.353112622 | 0.000982219 | 0.010660671 |
|  | *Ocrl* TRCN0000080956 |  | 31753 | 7622 | 0.240040311 | 5.85523E-05 | 0.00301123 |
| 37 | *Depdc1b* TRCN0000193930 | 2 of 5 | 10788 | 3655 | 0.338802373 | 0.012819557 | 0.054073014 |
|  | *Depdc1b* TRCN0000173585 |  | 282.2 | 60.81 | 0.215485471 | 0.022757969 | 0.077172251 |
| 38 | *Pik3r2* TRCN0000025088 | 2 of 4 | 1091 | 349.8 | 0.320623281 | 0.010271809 | 0.04545785 |
|  | *Pik3r2* TRCN0000025086 |  | 6190 | 1191 | 0.192407108 | 0.000155262 | 0.003153132 |
| 39 | *Arap3* TRCN0000106139 | 2 of 9 | 25110 | 7016 | 0.279410593 | 0.004305785 | 0.026069039 |
|  | *Arap3* TRCN0000295613 |  | 482.2 | 69.26 | 0.143633347 | 0.003181697 | 0.021026456 |
| 40 | *Arhgap20* TRCN0000097343 | 2 of 5 | 9550 | 1640 | 0.171727749 | 0.000155885 | 0.003153132 |
|  | *Arhgap20* TRCN0000097342 |  | 2840 | 330.5 | 0.116373239 | 0.000323461 | 0.004798012 |
| 41 | *Gmip* TRCN0000028298 | 2 of 5 | 12441 | 1963 | 0.157784744 | 6.77474E-05 | 0.00301123 |
|  | *Gmip* TRCN0000028244 |  | 286.4 | 42.96 | 0.15 | 0.00442051 | 0.026086001 |
| RhoGDIs |  |  |  |  |  |  |  |
| 42 | *Arhgdib* TRCN0000106180 | 2 of 5 | 52693 | 23071 | 0.437838043 | 0.024045777 | 0.080453915 |
|  | *Arhgdib* TRCN0000106181 |  | 71690 | 10746 | 0.149895383 | 0.00018072 | 0.00342214 |
|  | *Arhgdib* TRCN0000106183 |  | 19665 | 2443 | 0.124230867 | 4.4632E-05 | 0.00301123 |
|  | *Arhgdib* TRCN0000106182 |  | 3855 | 1074 | 0.278599222 | 0.000287869 | 0.004494796 |
