# Supplementary material for: An RNAi screen unravels the complexities of Rho GTPase networks in skin morphogenesis
Source: eLife. 2019 Sep 25;8:e50226. doi: 10.7554/eLife.50226 (PMC6768663; doi:10.7554/eLife.50226)
Supplement: Supplementary file 5. [file elife-50226-supp5.docx]

**Supplementary File 5.** Genes With ≥ Two shRNAs Showing an Absolute Enrichment or Depletion only in the Hair Follicle fraction

| Candidate | Gene | Mean In vitro | Mean E18.5 HF | Fold change | *P* value | q value |
| --- | --- | --- | --- | --- | --- | --- |
| Rho GTPases |  |  |  |  |  |  |
| 1 | *Rhou* TRCN0000287360 | 361.7 | 1731 | 4.785734034 | 2.56432E-05 | 0.001281156 |
|  | *Rhou* TRCN0000077506 | 1654 | 4142 | 2.504232164 | 0.007898817 | 0.022724639 |
|  | *Rhou* TRCN0000077505 | 635.8 | 1548 | 2.434727902 | 0.004731611 | 0.016449743 |
| 2 | *Rhob* TRCN0000077533 | 104333 | 47399 | 0.454304966 | 0.004463716 | 0.015827519 |
|  | *Rhob* TRCN0000077534 | 53638 | 9107 | 0.169786346 | 0.001368599 | 0.007427153 |
| 3 | *Rac3* TRCN0000065396 | 6251 | 2539 | 0.406175012 | 0.002363527 | 0.010320085 |
|  | *Rac3* TRCN0000065393 | 71947 | 27482 | 0.381975621 | 0.012764056 | 0.032181331 |
| 4 | *Rac1* TRCN0000055190 | 35603 | 13614 | 0.382383507 | 0.00649188 | 0.020046654 |
|  | *Rac1* TRCN0000055192 | 52705 | 15837 | 0.300483825 | 0.008017457 | 0.022724639 |
|  | *Rac1* TRCN0000055189 | 7963 | 1300 | 0.163255055 | 8.49622E-05 | 0.00180039 |
| RhoGEFs |  |  |  |  |  |  |
| 5 | *Arhgef40* TRCN0000121408 | 13354 | 6659 | 0.498652089 | 0.000265726 | 0.003093146 |
|  | *Arhgef40* TRCN0000121410 | 1889 | 500.1 | 0.26474325 | 0.000577728 | 0.004357437 |
| 6 | *Arhgef3* TRCN0000110049 | 14521 | 7214 | 0.496797741 | 0.001121287 | 0.006438355 |
|  | *Arhgef3* TRCN0000110047 | 13573 | 6043 | 0.445222132 | 0.002153773 | 0.009779889 |
|  | *Arhgef3* TRCN0000110046 | 7497 | 2913 | 0.388555422 | 0.03905341 | 0.07348316 |
| 7 | *Prex1* TRCN0000247271 | 432.3 | 211.3 | 0.488780939 | 0.017277536 | 0.039940279 |
|  | *Prex1* TRCN0000247272 | 170.4 | 41.87 | 0.245715962 | 0.000331545 | 0.003191108 |
|  | *Prex1* TRCN0000247273 | 565.6 | 226.5 | 0.400459689 | 0.020381513 | 0.044433398 |
| 8 | *Arhgef1* TRCN0000110053 | 18473 | 8991 | 0.486710334 | 0.004243924 | 0.015108369 |
|  | *Arhgef1* TRCN0000110054 | 35669 | 6249 | 0.175194146 | 0.000544886 | 0.004191829 |
|  | *Arhgef1* TRCN0000110050 | 94.42 | 24.84 | 0.263079856 | 0.017562982 | 0.040390321 |
| 9 | *Dock2* TRCN0000091247 | 2971 | 1445 | 0.486368226 | 0.009792336 | 0.026438881 |
|  | *Dock2* TRCN0000091246 | 12116 | 4060 | 0.33509409 | 0.00078306 | 0.005162396 |
| 10 | *Arhgef9* TRCN0000226313 | 1421 | 617.8 | 0.434764251 | 0.016007088 | 0.037688646 |
|  | *Arhgef9* TRCN0000258240 | 227.9 | 33.85 | 0.148530057 | 0.02090024 | 0.045148577 |
| 11 | *Mcf2* TRCN0000042654 | 17131 | 7327 | 0.427704162 | 0.00675353 | 0.020584389 |
|  | *Mcf2* TRCN0000174066 | 88.38 | 31.2 | 0.353021045 | 0.000277004 | 0.003093146 |
| 12 | *Fgd2* TRCN0000110044 | 20446 | 7706 | 0.376895236 | 0.030506741 | 0.061013482 |
|  | *Fgd2* TRCN0000110041 | 116.3 | 43.56 | 0.374548581 | 0.006389555 | 0.019814298 |
| 13 | *Dnmbp* TRCN0000329046 | 1289 | 483.4 | 0.375019395 | 0.048523147 | 0.086892558 |
|  | *Dnmbp* TRCN0000329105 | 1110 | 392.6 | 0.353693694 | 0.005768736 | 0.018468255 |
| 14 | *Als2* TRCN0000041030 | 76865 | 27060 | 0.352045795 | 0.001432711 | 0.007500664 |
|  | *Als2* TRCN0000041032 | 40998 | 6743 | 0.164471438 | 0.000180149 | 0.002419948 |
| 15 | *Dock10* TRCN0000251515 | 4730 | 1624 | 0.343340381 | 0.00015923 | 0.002285726 |
|  | *Dock10* TRCN0000251513 | 288.6 | 31.93 | 0.110637561 | 0.006105942 | 0.019270525 |
| 16 | *Dock11* TRCN0000217207 | 30857 | 9835 | 0.318728327 | 0.002974017 | 0.01176389 |
|  | *Dock11* TRCN0000200778 | 19255 | 3378 | 0.175434952 | 0.000168043 | 0.002369345 |
| 17 | *Trio* TRCN0000254106 | 1095 | 320.4 | 0.29260274 | 0.002257137 | 0.010113594 |
|  | *Trio* TRCN0000254107 | 756.5 | 113.1 | 0.149504296 | 0.001898443 | 0.00898731 |
| 18 | *Plekhg1* TRCN0000251310 | 5960 | 1669 | 0.280033557 | 0.014826608 | 0.03585783 |
|  | *Plekhg1* TRCN0000251309 | 625.9 | 130.9 | 0.20913884 | 0.002734308 | 0.011163 |
| RhoGAPs |  |  |  |  |  |  |
| 19 | *Arhgap36* TRCN0000283769 | 336.1 | 909.6 | 2.7063374 | 0.009854424 | 0.026487763 |
|  | *Arhgap36* TRCN0000283770 | 143.8 | 318.4 | 2.21418637 | 0.004888081 | 0.016861985 |
| 20 | *Arhgap8* TRCN0000097337 | 80511 | 37336 | 0.463737874 | 0.032237614 | 0.063197084 |
|  | *Arhgap8* TRCN0000097338 | 6330 | 465.3 | 0.073507109 | 3.15438E-05 | 0.001281156 |
| 21 | *Arhgap23* TRCN0000179417 | 38523 | 15498 | 0.402305116 | 0.001189521 | 0.006662336 |
|  | *Arhgap23* TRCN0000184726 | 3653 | 1359 | 0.372022995 | 0.001576208 | 0.007970597 |
| 22 | *Chn1* TRCN0000112395 | 44355 | 16051 | 0.361875775 | 0.001424229 | 0.007500664 |
|  | *Chn1* TRCN0000112396 | 4065 | 709.4 | 0.174514145 | 1.59982E-05 | 0.001281156 |
| 23 | *Stard13* TRCN0000106252 | 33023 | 11916 | 0.360839415 | 0.000395262 | 0.003463678 |
|  | *Stard13* TRCN0000106254 | 64164 | 18709 | 0.291580949 | 0.000839162 | 0.005401014 |
| 24 | *Arhgap15* TRCN0000197579 | 12542 | 4279 | 0.341173657 | 0.001923513 | 0.00901014 |
|  | *Arhgap15* TRCN0000176672 | 63607 | 2472 | 0.038863647 | 0.001737919 | 0.00858017 |
| 25 | *Depdc7* TRCN0000191595 | 9004 | 3064 | 0.340293203 | 0.014811764 | 0.03585783 |
|  | *Depdc7* TRCN0000200619 | 56502 | 8383 | 0.14836643 | 0.00017695 | 0.002419948 |
| 26 | *Arhgap11a* TRCN0000193496 | 48199 | 15315 | 0.317745181 | 0.005449449 | 0.017794787 |
|  | *Arhgap11a* TRCN0000193495 | 5506 | 775.6 | 0.140864511 | 1.77487E-05 | 0.001281156 |
