# Supplementary material for: An RNAi screen unravels the complexities of Rho GTPase networks in skin morphogenesis
Source: eLife. 2019 Sep 25;8:e50226. doi: 10.7554/eLife.50226 (PMC6768663; doi:10.7554/eLife.50226)
Supplement: Supplementary file 7. [file elife-50226-supp7.docx]

| **Key Resources Table** | | | | |
| --- | --- | --- | --- | --- |
| **Reagent type**  **(species) or**  **resource** | **Designation** | **Source or Reference** | **Identifiers** | **Additional information** |
| experimental model, mouse strain | Mouse: CD1/ICR | Charles River | 022 |  |
| experimental model, mouse strain | Mouse: K14-rtTA | Fuchs Lab |  |  |
| experimental model, mouse strain | Mouse: K14-Actin-GFP | Fucsh Lab |  |  |
| cell line  (*M. musculus*) | CD1 derived primary keratinocytes | This paper |  | Cell line generated and maintained as in Blanpain et al. 2004 |
| cell line  (*M. musculus*) | K14rtTA derived primary keratinocytes | This paper |  | Cell line generated and maintained as in Blanpain et al. 2004 |
| antibody | anti-ARGEF7 | Millipore | Cat#:  07-1450-I  RRID:  AB_1586904 | IF (1:200) WB (1:1000) |
| antibody | anti-human and mouse CD49f-PE/Cy7  (rat monoclonal) | BioLegend | Cat#:  313622  RRID: AB_2561705 | FACS  (1:1000) |
| antibody | anti-mouse CD31-Biotin  (rat monoclonal) | BioLegend | Cat#:  102504  RRID:  AB_312911 | FACS (1:200) |
| antibody | anti-mouse CD45-Biotin  (rat monoclonal) | BD | Cat#:  553077  RRID:  AB_394607 | FACS (1:200) |
| antibody | anti-mouse CD117-Biotin  (rat monoclonal) | BioLegend | Cat#:  105804  RRID:  AB_313213 | FACS (1:200) |
| antibody | anti-mouse CD140a-Biotin  (rat monoclonal) | BioLegend | Cat#:  135910  RRID:  AB_2043974 | FACS (1:200) |
| antibody | all species Streptavidin-FITC | BioLegend | Cat#:  405202 | FACS (1:500) IF(1:1000) |
| antibody | all species Streptavidin-APC/Cy7 | BD | Cat#:  554063  RRID:  AB_10054651 | FACS (1:500) |
| antibody | anti-mouse CELSR1  (polyclonal guinea pig) | Fuchs Lab |  | IF (1:250) |
| antibody | anti-mouse E-CADHERIN  (rat monoclonal) | Fuchs Lab gift from Dr Takeichi |  | IF (1:50) |
| antibody | all species anti-GFP  (chicken polyclonal) | Abcam | Cat#:  Ab13970  RRID:  AB_300798 | IF (1:2000)  WB (1:10 000) |
| antibody | anti-mouse Keratin 5  (guinea pig polyclonal) | Fuchs Lab |  | IF (1:500) |
| antibody | anti-mouse Keratin 6  (guinea pig polyclonal) | Fuchs Lab |  | IF (1:1000) |
| antibody | anti-mouse Keratin 10  (rabbit polyclonal) | Covance | Cat#:  PRB-159P  RRID:  AB_291580 | IF (1:500) |
| antibody | anti-mouse LORICRIN  (rabbit polyclonal) | Covance | Cat#:  PRB-145P  RRID:  AB_292095 | IF (1:2000) |
| antibody | anti-human, mouse, rat myosin light chain 2  (rabbit polyclonal) | Cell Signaling | Cat#:  3672  RRID:  AB_10692513 | WB (1:1000) |
| antibody | anti-phopho human, mouse Myosin light chain 2 ^Thr18/Ser19^  (rabbit polyclonal) | Cell Signaling | Cat#:  3674  RRID:  AB_2147464 | IF (1:200)  WB (1:1000) |
| antibody | all species anti-MYC  (rabbit monoclonal) | Cell Signaling | Cat#:  2278  RRID:  AB_490778 | IF (1:200)  WB (1:1000) |
| antibody | anti-chicken, human, mouse rabbit NCAM  (rabbit polyclonal) | Millipore | Cat#:  AB5032  RRID:  AB_2291692 | IF (1:200) |
| antibody | anti-human, mouse, rat monkey, guinea pig PAK1/2/3  (rabbit polyclonal) | Cell signaling | Cat#:  2604  RRID:  AB_2160225 | WB (1:1000) |
| antibody | anti-human, mouse, rat, monkey, guinea pig PAK1  (rabbit polyclonal) | Cell signaling | Cat#:  2602  RRID:  AB_330222 | WB (1:1000) |
| antibody | anti-human, mouse, rat, monkey, guinea pig PAK2  (rabbit polyclonal) | Cell signaling | Cat#:  2608  RRID:  AB_2283388 | WB (1:1000) |
| antibody | anti-phospho human mouse guinea pig PAK1^Ser144^/PAK2 ^Ser141^  (rabbit polyclonal) | Cell signaling | Cat#:  2606  RRID:  AB_2299279 | WB (1:1000) |
| antibody | anti-mouse P-CADHERIN  (goat polyclonal) | R&D | Cat#:  AF761  RRID:  AB_355581 | IF (1:400) |
| antibody | anti-human, mouse RHOU  (rabbit polyclonal) | OriGene | Cat#:  TA344077 | IF (1:1000)  WB (1:10 000) |
| antibody | all species anti-RFP  (rat monoclonal) | Chromotek | Cat#:  5f8  RRID:  AB_2336064 | IF (1:1000) |
| antibody | anti-bovine, canine, mouse, rat, turkey, human, chicken, frog VINCULIN  (mouse monoclonal) | Sigma | Cat#:  V9131  RRID:  AB_477629 | IF (1:200) |
| antibody | anti- bovine, rat, yeast, human, mouse, chicken, gungi, amphibian TUBULIN  (mouse monoclonal) | Sigma | Cat#:  T5168  RRID:  AB_477579 | WB (1:10 000) |
| antibody | anti-rabbit AF488 conjugated secondary  (donkey polyclonal) | Jackson ImmunoResearch | Cat#:  711-545-152  RRID:  AB_2323584 | IF (1:1000) |
| antibody | anti-rabbit AF546 conjugated secondary  (donkey polyclonal) | Jackson ImmunoResearch | Cat#:  711-165-152  RRID:  AB_2307443 | IF (1:1000) |
| antibody | anti-rabbit AF647 conjugated secondary  (donkey polyclonal) | Jackson ImmunoResearch | Cat#:  711-605-152  RRID:  AB_2492288 | IF (1:1000) |
| antibody | anti-guinea pig AF488 conjugated antibody  (donkey polyclonal) | Jackson ImmunoResearch | Cat#:  706-545-148  RRID:  AB_2340472 | IF (1:1000) |
| antibody | anti-rat AF488 conjugated antibody  (donkey polyclonal) | Jackson ImmunoResearch | Cat#:  712-546-153  RRID:  AB_2340686 | IF (1:1000) |
| antibody | anti-rat RRX conjugated antibody  (donkey polyclonal) | Jackson ImmunoReserach | Cat#:  712-295-153  RRID:  AB_2340676 | IF (1:1000) |
| antibody | anti-rat AF647 conjugated antibody  (donkey polyclonal) | Jackson ImmunoResearch | Cat#:  712-605-153  RRID:  AB_2340694 | IF (1:1000) |
| antibody | anti-chicken AF488 conjugated antibody  (donkey polyclonal) | Jackson ImmunoResearch | Cat#:  703-545-155  RRID:  AB_2340375 | IF (1:1000) |
| antibody | anti-goat AF647 conjugated antibody  (donkey polyclonal) | Jackson ImmunoResearch | Cat#:  705-605-003 | IF (1:1000) |
| antibody | TrueBlot anti-rabbit IgG HRP | Rockland | Cat#:  18-8816-33  RRID:  AB_2610848 | WB (1:10 000) |
| antibody | TrueBlot anti-mouse IgG HRP | Rockland | Cat#:  18-8814-33  RRID:  AB_2610844 | WB (1:10 000) |
| sequenced-based reagent | qPCR primers.  *See Supplementary file 2 for a complete list* | This study (Eurofinsgenomis) | qPCR primers |  |
| sequenced-based reagent | TRC library clones.  *See Supplementary File 1 and Supplementary File 2 for a complete list* | Fuchs Lab/Sigma | TRC library clones |  |
| recombinant DNA reagent | *pLKO.1* TRC Cloning Vector  (plasmid) | Addgene | Cat#:  10878  RRID:  Addgene_10878 |  |
| recombinant DNA reagent | *pCMV6-Rhou*  (plasmid) | Origene | Cat#:  MR219421 |  |
| recombinant DNA reagent | *mycBioID2-pBABE-puro*  (plasmid) | Addgene | Cat#:  80900  RRID:  Addgene_80900 |  |
| recombinant DNA reagent | *LV-U6-PGK-H2B-RFP*  (plasmid) | Fuchs Lab |  |  |
| recombinant DNA reagent | *LV-U6-PGK-H2B-GFP*  (plasmid) | Fuchs Lab |  |  |
| recombinant DNA reagent | *LV-Tre-pgk-H2B-RFP*  (plasmid) | Fuchs Lab |  |  |
| recombinant DNA reagent | *LV-Tre-MycRhou-pgk-H2B-RFP*  (plasmid) | This paper |  |  |
| recombinant DNA reagent | *LV-Tre-MycBioID2GFP-pgk-H2B-RFP*  (plasmid) | This paper |  |  |
| recombinant DNA reagent | *LV-Tre-MycBioID2Rhou-pgk-H2B-RFP*  (plasmid) | This paper |  |  |
| chemical compound, drug | Doxycycline | Sigma | Cat#:  D9891 |  |
| chemical compound, drug | Puromycin Dihydrochloride | ThermoFisher | Cat#:  A1113803 |  |
| chemical compound, drug | TRI Reagent | Sigma | Cat#:  T3934 |  |
| chemical compound, drug | Biotin | Sigma | Cat#:  B4501 |  |
| chemical compound, drug | 16% Paraformaldehyde Solution | Electron Microscopy Science | Cat#:  15700 |  |
| peptide, recombinant protein | Human Plasma Fibronectin Purified Protein | Millipore | Cat#:  FC010 |  |
| commercial assay or kit | Direct-zol RNA mini prep kit | Zymo Research | Cat#:  R2050 |  |
| commercial assay or kit | Superscript VILO cDNA Synthesis kit | Invitrogen | Cat#:  11754050 |  |
| commercial assay or kit | QIAquick Gel Extraction kit | Qiagen | Cat#:  28704 |  |
| commercial assay or kit | DNA Clean & Concentrator^TM^-5 column | Zymo Research | Cat#:  D4031 |  |
| commercial assay or kit | TUNEL Alexa Fluor^TM^ 647 Imaging Assay for microscopy | Life Technologies | Cat#:  C10247 |  |
| commercial assay or kit | DNeasy Blood & Tissue Kit | Qiagen | Cat#:  69504 |  |
| commercial assay or kit | Click-it Edu Alexa Fluor 647 Imaging kit | Life Technologies | Cat#:  C10340 |  |
| commercial assay or kit | SYBR Green PCR Master Mix | Applied Biosystems | Cat#:  4367659 |  |
| commercial assay or kit | Effectene Reagent | Qiagen | Cat#:  301425 |  |
| software, algorithm | Graphpad Prism 8 | Graphpad.com |  |  |
| software, algorithm | Fiji (Image J) | (<https://fiji.sc/>) | RRID:  SCR_003070 |  |
| software, algorithm | Adobe Illustrator | Adobe.com |  |  |
| software, algorithm | R studio | Rstudio.com |  |  |
| software, algorithm | DESeq2 in R | Love et al., 2014 |  |  |
| software, algorithm | DAVID | (https//david.ncifcrf.gov) | Huang et al., 2008  RRID:  SCR_001881 |  |
| software, algorithm | FlowJo Software | BD Biosciences |  |  |
| software, algorithm | FACS DiVa software | BD Biosciences |  |  |
| software, algorithm | CellProfiler^TM^ | Jones et al., BMC Bioinformatics 2008  (www.cellprofiler.org) | RRID:  SCR_007358 |  |
| other | Phusion High-Fidelity DNA Polymerase | NEB | Cat#:  M0530 |  |
| other | Clarity^TM^ Western ECL Substrate | BioRad | Cat#:  179-5060 |  |
| other | ProLong^TM^ Gold antifade reagent with DAPI | TermoFisher | Cat#:  36935 |  |
| other | Alexa Fluor^TM^ 647 Phalloidin | Thermo Fisher | Cat#:  22287 |  |
| other | OCT Compound Tissue Tek | VWR | Cat#:  25608-930 |  |
| other | Dispase | Gibco | Cat#:  17105-041 |  |
| other | Trypsin-EDTA 0.25% | Gibco | Cat#:  25200056 |  |
| other | Versene | Gibco | Cat#:  13151014 |  |
| other | cOmplete^TM^ Protease Inhibitor Cocktail | Sigma | Cat#:  11697498001 |  |
| other | RIPA lysis and extraction buffer | ThermoFisher | Cat#:  89900 |  |
| other | phosSTOP^TM^ | Sigma | Cat#:  4906845001 |  |
| other | Millicell EZ SLIDE 4 well glass slide | Millipore | Cat#:  PEZGSO416 |  |
| other | ZebaTM Spin Desaltin Columns (7K MWCO for 10 ml) | ThermoFisher | Cat#:  89893 |  |
| other | Dynabeads^TM^MyOne^TM^Streptavidin T1 | ThermoFisher | Cat#:  65601 |  |
| other | DynaMag^TM^-2 Magnet | ThermoFisher | Cat#:  12321D |  |
| other | NuPAGE^TM^ 4-12% Bis-Tris Protein Gel | ThermoFisher | Cat#:  NP0321 |  |
